# EPURE Transplant (Eplerenone in Patients Undergoing Renal Transplant) study: study protocol for a randomized controlled trial

**DOI:** 10.1186/s13063-018-2956-1

**Published:** 2018-10-30

**Authors:** Sophie Girerd, Luc Frimat, Didier Ducloux, Yannick Le Meur, Christophe Mariat, Bruno Moulin, Christiane Mousson, Philippe Rieu, Nassim Dali-Youcef, Ludovic Merckle, Xavier Lepage, Patrick Rossignol, Nicolas Girerd, Frédéric Jaisser

**Affiliations:** 1 Transplant Unit, Nephrology Department, Nancy University Hospital, Lorraine University, Vandoeuvre-lès-Nancy, France; 2 INSERM U1116, Clinical Investigation Center, Lorraine University, Vandoeuvre-lès-Nancy, France; 3 INI-CRCT (Cardiovascular and Renal Clinical Trialists) F-CRIN network, Nancy, France; 4 Transplant Unit, Nephrology Department, Besançon University Hospital, Bourgogne Franche-Comté University, Besançon, France; 5 Department of Nephrology, Brest University Hospital, Brest University, Brest, France; 6 Transplant Unit, Nephrology Department, Saint-Etienne University Hospital, Jean Monnet University, Saint-Etienne, France; 7 Nephrology and Transplantation Department, Strasbourg University Hospital, Strasbourg University, Strasbourg, France; 8 Transplant Unit, Nephrology Department, Dijon University Hospital, Bourgogne Franche-Comté University, Dijon, France; 9 Transplant Unit, Nephrology Department, Reims University Hospital, Reims Champagne-Ardenne University, Reims, France; 10 Department of Biochemistry and Molecular Biology, Strasbourg University Hospital, Strasbourg University, Strasbourg, France; 11 Department of functional Genomics and Cancer, Institut de Génétique et de Biologie Moléculaire et Cellulaire (IGBMC)/ CNRS UMR 7104/ INSERM U 964/ Strasbourg University, 1 rue Laurent Fries, 67404 Illkirch, France; 12 INSERM, UMRS 1138, Team 1, Centre de Recherche des Cordeliers, Pierre et Marie Curie University, Paris Descartes University, Paris, France

**Keywords:** Mineralocorticoid receptor antagonist, Ischemia/reperfusion lesions, Kidney transplantation, Expanded criteria donor, Randomized controlled trial

## Abstract

**Background:**

Despite advances in immunosuppressive therapy, kidney graft survival has failed to improve during the last decades. Ischemia/reperfusion injury (IRI) is one of the main pathophysiological mechanisms underlying delayed graft function, which is associated with poor long-term graft survival. Due to organ shortage, the proportion of grafts from expanded criteria donors (ECDs) is ever growing. These grafts may particularly benefit from IRI prevention. In preclinical models, mineralocorticoid receptor antagonists (MRAs) have been shown to efficiently prevent IRI. This study aims to assess the effect of MRA administration in the early phase of kidney transplantation (KT) among recipients of ECD grafts on mid-term graft function.

**Methods/design:**

This is a multicenter, double-blind, placebo-controlled, randomized clinical trial. Patients on hemodialysis and undergoing a single or a dual KT from an ECD will be eligible for inclusion. We plan to randomize 132 patients. Included patients will be randomized (1:1) to receive either eplerenone 25 mg every 12 h during 4 days (the first dose being administered just prior to KT) or placebo. The primary outcome is graft function at 3 months, assessed by glomerular filtration rate (GFR, in mL/min/1.73m^2^) measured using iohexol clearance. Secondary outcomes include (1) proportion of patients with either dialysis dependency or a GFR < 30 mL/min/1.73m^2^ at 3 months, (2) proportion of patients with immediate, slow, or delayed graft function, (3) proteinuria at 3 months, (4) occurrence of hyperkalemia during the first week following KT, (5) length of hospital stay for the KT, and (6) occurrence of biopsy-proven acute rejection in the first 3 months following KT. Estimated GFR, graft, and patient survival will also be collected at 1, 3, and 10 years via the national database of organ recipients.

**Discussion:**

Improvement of ECD grafts is a public health priority, since better ECD outcomes could eventually limit organ shortage. MRA administration in the early phase of KT may prevent IRI and subsequently improve mid-term graft function. The trial will also assess the safety of MRA administration in this population, primarily the absence of threatening hyperkalemia.

**Trial registration:**

ClinicalTrials.gov, NCT02490904. Registered on 1 July 2015.

**Electronic supplementary material:**

The online version of this article (10.1186/s13063-018-2956-1) contains supplementary material, which is available to authorized users.

## Background

For patients with end-stage renal disease (ESRD), kidney transplantation (KT) is the treatment of choice in order to improve survival [1, 2], quality of life [3, 4], and health costs [3, 5]. Nevertheless, KT faces organ shortage, leading to a greater use of kidneys from expanded criteria donors (ECDs), which are more susceptible to deleterious ischemia/reperfusion (I/R) lesions [6–8]. I/R injury (IRI) is one of the principal pathophysiological mechanisms underlying delayed graft function (DGF), which is associated with poor long-term graft survival [9]. Episodes of IRI [10] and acute kidney injury (AKI) [11, 12] can lead to chronic kidney disease (CKD) with decreased glomerular filtration rate (GFR).

The use of preclinical models has identified several therapeutic strategies to prevent IRI, although these potential therapeutic avenues have been poorly translated to the human setting [10]. Accumulating evidence during the past decade suggests that blocking the mineralocorticoid receptor (MR) may represent a useful strategy to protect against I/R-related lesions. Preclinical studies in rodents and pigs have demonstrated a beneficial effect of MR antagonists (MRAs) in preventing acute IRI and mid-term GFR following AKI [13–17]. Of note, a pilot clinical trial on living-donor renal transplantation [18] demonstrated a beneficial effect of spironolactone on renal oxidative stress when administered to recipients 1 day before and 3 days after KT. However, this trial was not associated with improved short-term renal function, since renal function was already good in the placebo group, as expected in living-donor transplantation. However, there has yet to be an assessment of the effect of MRA in ECD kidney grafts, which usually experience a much lower GFR than living-donor grafts.

### Hypothesis

Our hypothesis is that MRA administration during the perioperative period of KT can limit IRI, thereby resulting in improved graft function in the setting of KT with ECD.

## Methods/design

### Study design

This is a multicenter, double-blind, placebo-controlled, randomized clinical trial. Patients on hemodialysis and undergoing a single or a dual KT from an ECD will be eligible for inclusion after verification of the inclusion criteria listed in the following section. Included patients will be randomized (1:1) to receive either eplerenone 25 mg every 12 h during 4 days (the first dose being administered just prior to KT) or placebo.

### Participants

#### Inclusion criteria

Graft recipients on chronic hemodialysis aged 18 or older will be eligible for inclusion if they:

- Sign the informed consent for study participation
- Are about to receive a single or a dual kidney graft from an ECD (brain-dead donor aged > 60 years or aged 50–59 years with 2+ characteristics among vascular cause of death, medical history of hypertension or serum creatinine > 130 μmol/L) regardless of machine perfusion use and graft rank
- Are affiliated with a medical care system.

#### Exclusion criteria

Graft recipients will be excluded if they have any of the following conditions:

- Are about to receive multiple organ transplantation involving organs other than kidney
- Are on chronic peritoneal dialysis
- Are not yet on dialysis and are about to receive a preemptive KT
- Have a known hypersensitivity or allergy to eplerenone, its excipients, or lactose
- Have severe cirrhosis (Child-Pugh class C)
- Are receiving potent CYP3A4 inhibitors (e.g., itraconazole, ketoconazole, ritonavir, nelfinavir, clarithromycin, telithromycin, and/or nefazodone)
- Are receiving a graft from a donor treated with an MRA (spironolactone or eplerenone)
- Have a known hypersensitivity or allergy to iodine contrast products (since iohexol will be used for GFR quantification)
- Have thyrotoxicosis,
- Have undergone human leukocyte antigen (HLA) desensitization
- Are pregnant or do not use an effective means of contraception (for women)
- Are a patient under judicial protection
- Are a patient under tutorship or legal guardianship
- Are already participating in another medical research study.

#### Participant recruitment

Patients will be included during their hospitalization for KT. The study will be explained to the patients fulfilling the inclusion criteria at admission. Patients will receive a written information document and will have a time delay to decide if they wish to be included in the study (see Additional files 2 and 3). Note that this time delay may be shorter when compared to clinical trials performed in other settings, as the KT may be performed within a few hours after the patient’s admission. There is no minimal prespecified time delay: it is the investigator’s responsibility to ensure that patients had sufficient time to rightfully decide to participate in the study. Inclusion and randomization will be performed when KT scheduling is confirmed, i.e., in the absence of any contraindication for the KT, either related to the recipient, to the graft, or to an immunological issue based on daily or virtual cross-match.

#### Randomization

Randomization will be provided via the Cleanweb™ system based on a prespecified randomization list using blocks, stratified on centers and machine perfusion use.

### Interventions

#### Timing of administration of the study treatment

**Fig. 1:**
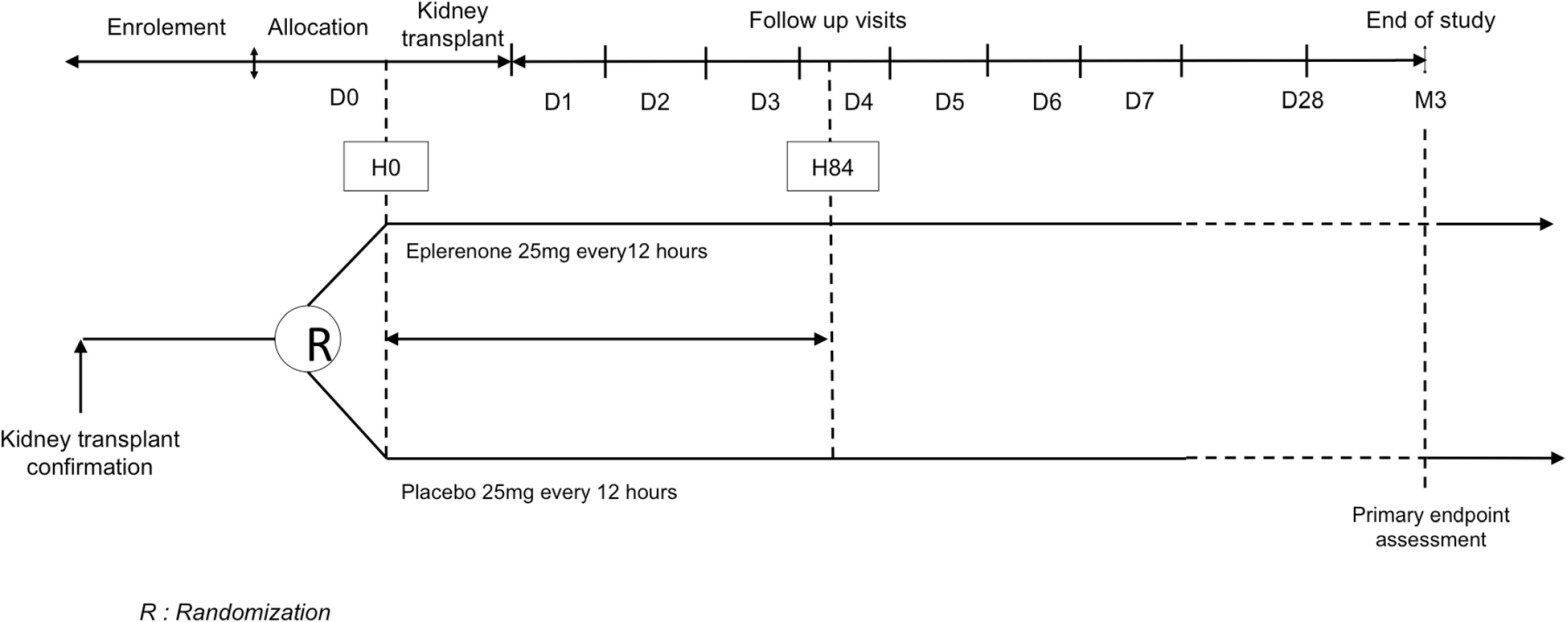
Schedule of enrollment, intervention, and assessments

The first administration of the study treatment (eplerenone 25 mg or placebo) will be performed within 2 h prior to patient departure to the operating room, usually at the same time as the first administration of immunosuppressive therapy (H0). See Fig. 1. Of note, treatment will be initiated only if a dosage of serum potassium ≤5 mmol/L within the previous 12 h is available.

The remaining study treatment units will be given every 12 h for 4 days; thus, eight treatment units (H0, H12, H24, H36, H48, H60, H72, H84) will ultimately be administered to the patients.

#### Posology change

No change in treatment dose is planned. If patients experience clinically significant hyperkalemia, treating physicians will apply local protocols (e.g., administer potassium-binding resins, correct metabolic acidosis, and/or schedule a dialysis session).

#### Premature discontinuation of the study treatment

Study treatment will be discontinued if allergy to eplerenone or excipients is suspected. If the study treatment is discontinued, patients will be followed until the end of the study period. Hyperkalemia will not trigger treatment discontinuation.

#### Appropriate administration of treatment units

Blister packs will be returned to the hospital pharmacy to verify treatment administration.

#### Unblinding modalities

Unblinding will be authorized in patients for whom knowing treatment allocation will eventually lead to a modification in management and care. Investigators can call the 24/7 phone number of the Poison Control Center in charge of the study to request unblinding.

#### Concomitant medication

During the study treatment administration phase (i.e., 4 days), patients cannot receive angiotensin-converting enzyme inhibitors (ACEi) and/or angiotensin receptor blockers (ARBs) and/or renin inhibitors and/or nonsteroidal anti-inflammatory drugs (NSAIDs) and/or potassium-sparing diuretics and/or strong CYP3A inhibitors (e.g., ketoconazole, itraconazole, nefazodone, clarithromycin, ritonavir, nelfinavir, etc.).

Concomitant use of cyclosporine or tacrolimus is allowed.

If needed, potassium supplementation is allowed in patients with hypokalemia due to hypokalemic polyuria following graft recovery or loop diuretic use. A close monitoring of serum potassium levels and renal function will be mandatory.

The choice of immunosuppressive therapy modalities and use of dialysis after transplant is left to the discretion of the treating physicians, and the participating centers may use their usual standard practices. The study treatment is considered as a supplementary intervention to improve graft function irrespective of the immunosuppressive regimen.

### Study outcomes

The primary outcome of the study is graft function at 3 months assessed by GFR (mL/min/1.73m^2^) using iohexol clearance. Two hours after intravenous injection of 3.235 × 10^6^ μg iohexol, 5-mL blood samples will be collected every 30 min for 2.5 h. After centrifugation at room temperature (2500 *g* for 10 min), the plasma will be stored at − 20° until high performance liquid chromatography (HPLC) measurement of iohexol concentration using an ODS C18 ultrasphere Beckman Coulter column 5 μm × 4.6 mm × 250 mm and an ultraviolet spectrophotometer detector at 240 nm (Shimadzu France, Champs s/Marne, France) as previously reported [19]. The plasma clearance of iohexol will be determined by the one-compartment model from the area under the curve of iohexol elimination from the plasma with the formula: Clearance (Cl) = dose/area under curve (AUC). The area will be corrected for the early distribution phase according to Bröchner-Mortensen; the corrected Cl calculated as (0.990778 × Cl) – (0.001218 × Cl) [20] will be used as the actual primary endpoint.

In patients with primary non-function (defined by the necessity for dialysis at 3 months post-KT), an arbitrary value of 5 mL/min will be used as GFR*.*

Secondary outcomes include:

1. The proportion of patients with either dialysis dependency or a GFR < 30 mL/min/1.73m^2^ at 3 months
2. The time delay for graft recovery assessed by:
  The proportion of patients with a DGF defined by the necessity of one or more dialysis sessions during the 7 days following KT
  The proportion of patients with immediate (defined by a serum creatinine < 30 mg/L at 7 days post-KT), slow (defined by a serum creatinine > 30 mg/L at 7 days post-KT without dialysis requirement), or DGF (defined by the necessity of one or more dialysis sessions during the 7 days following KT)
3. Twenty-four hour proteinuria and microalbuminuria levels at 3 months
4. The occurrence of hyperkalemia > 6 mmol/L during the first week following KT
5. The length of hospital stay for the KT (days)
6. The occurrence of biopsy-proven acute rejection in the first 3 months following KT

In addition, estimated GFR (eGFR, using the Chronic Kidney Disease-Epidemiology Collaboration (CKD-EPI) formula), graft, and patient survival data will be collected at 1, 3, and 10 years via the French national database of organ recipients (CRISTAL, Agence de la Biomédecine).

### Study visits

**Fig. 2:**
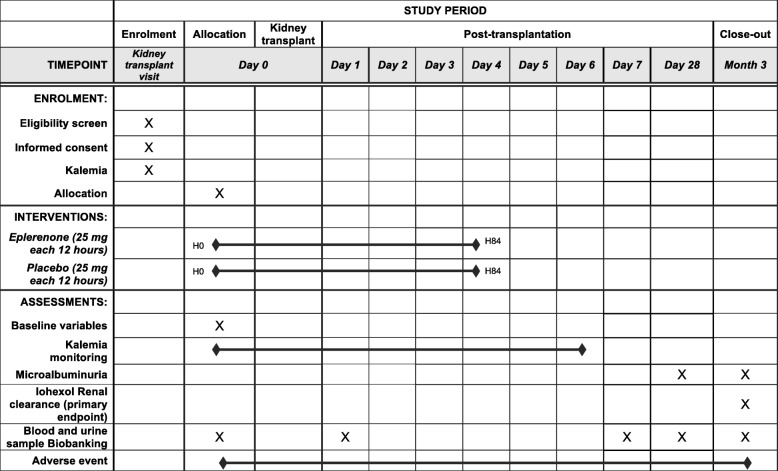
Randomization, timing of administration of the study treatment, and primary outcome measurement

As presented in Fig. 2, study visits will be performed daily until day 7, then at day 28, and finally at 3 months post-KT for the measurement of the primary outcome. Blood and urinary biological samples retrieved for biobanking purposes will be drawn at day 0 (randomization), day 1, day 7, day 28, and at 3 months.

### Adverse effects

The potassium level will be closely monitored throughout the treatment administration period. The serum potassium level must be verified as being ≤ 5 mmol/L in the 12 h prior to the first administration of eplerenone or placebo. Thereafter, the serum potassium level will be measured 6 h after the end of vascular clamping and subsequently twice a day until 48 h after the last administration of treatment (eplerenone or placebo). Thereafter, serum potassium levels will be monitored as typically performed in usual care.

Moreover, patients included in the study will receive the usual care performed during the post-operative KT period, including clinical workups (i.e., measurement of blood pressure, heart rate, and diuresis) as well as biological workups (serum creatinine, hemogram). All adverse events related to iohexol clearance will also be recorded.

Recording of serious adverse events (SAEs) will conform to Good Clinical Practice standards and will be in accordance with French regulation. Suspected unexpected SAEs/reactions will be reported by the attending physician to the sponsor immediately upon first awareness of their existence. It will be the responsibility of the investigators to collect all adverse events (either serious or non-serious) in the case report form (CRF) of the trial, during the follow-up of the patient. Adverse events (either serious or non-serious) will be collected until 48 h after the 3 months visit of the study. Every hyperkalemia episode leading to a supplementary dialysis session will be considered as an SAE. The half-life of eplerenone is short (4 h), and its administration two times a day (b.i.d.) allows its easy discontinuation in case of a non-anticipated adverse event.

### Statistical analysis plan

#### Sample size and power calculation

According to French statistical data (https://www.agence-biomedecine.fr/annexes/bilan2016/donnees/organes/06-rein/synthese.htm), the mean eGFR at 3 months among patients receiving a kidney graft from an ECD is 42 mL/min. Inclusion of 126 patients will allow identifying of a difference of 7 mL/min/1.73 m^2^ between the two groups (42 mL/min/1.73 m^2^ in the placebo group and 49 mL/min/1.73 m^2^ in the eplerenone group), with a standard deviation of 14 mL/min, a two-sided alpha risk of 5%, and a beta risk of 20% (power 80%). Since it is anticipated that 5% of patients could be included but not randomized or could withdraw their consent, we will target the inclusion of 132 patients.

#### Statistical analysis

The primary outcome, which is to compare the mean measured GFR between the two groups, will be analyzed by a *t* test for independent samples, after verifying the normality of GFR distribution in each group. In case of non-normality, the non-parametric Mann-Whitney test will be used. An analysis adjusted on stratification factors will then be performed to avoid clustering effects arising from stratification [21].

Secondary outcomes 1, 2, 4, and 6 will be analyzed by a chi-squared test. Secondary outcomes 3 and 5 will be analyzed with a non-parametric Mann-Whitney test for independent groups, given that the distribution of proteinuria levels and length of hospital stay are most likely non-normal.

In the event of clinically significant differences between baseline characteristics of the patients of the two groups, possibly observed despite randomization due to the moderate number of patients in this trial, some explanatory analyses adjusted for these parameters and on stratification factors will be performed and presented in the publication. Linear regression will be used for continuous variables and logistic regression for dichotomous variables.

The results of the trial will be reported according to the Consolidated Standards of Reporting Trials (CONSORT) Statement (see Additional file 1).

### Ethical issues

#### Ethics Committee approval

This study follows the principles of the Declaration of Helsinki and is in accordance with Good Clinical Practice and French regulation. The trial has been approved by the French Health Authorities (Agence nationale de la sécurité du médicament et des produits de santé: ANSM) and the appropriate Ethics Committee (Comité de Protection des Personnes: CPP Est III).

#### Information and consent forms

The patients are included on the basis of written information and upon signing an informed consent form (see Additional files 2 and 3). The informed consent form specifies that the patient can withdraw from the study at any time without any prejudice with regard to current or future medical treatment.

#### Interim analyses and study termination rules

An independent Data Safety and Monitoring Board (DSMB) comprising four international experts in kidney transplantation, biostatistics, and pharmacology (Prof. M. Kessler, Prof. D. Anglicheau, Dr. E. Gayat, and Dr. S. Crepin) will be responsible for ensuring the safety of the trial and for monitoring the progress of the research in accordance with the protocol. SAEs will be monitored during DSMB sessions, blinded for the randomization arm. No interim analysis is planned. The DSMB may decide to terminate the trial early for the following reasons: detection of adverse events, poor data quality, low levels of implementation, fraud, or new information which would rule the trial unnecessary, futile, or unethical.

### Data quality and regulatory issues

#### Monitoring of the study

The sponsor of the study — the Nancy University Hospital (Direction de la recherche, CHRU Nancy, Rue du Morvan, 54500 Vandoeuvre les Nancy; phone: 33 3.83.15.52.85, fax: 33 3.83.15.74.51) — will monitor (via quality control and audits) the study in all sites to ensure that compliance with the protocol and applicable regulations is maintained and that data are collected in a timely, accurate, and complete manner. All SAEs will be reported to the French regulatory agency (Agence nationale de la sécurité du medicament et des produits de santé: ANSM). The sponsor will provide yearly security reports to the French regulatory agency and the Ethics Committee.

#### Data management of the study

Clinical data management is performed at the Nancy Clinical Investigation Center using the Ennov Clinical® v7.5 Electronic Data Capture solution. Individual and secured access is provided to remote users who performed the data entry on the electronic CRF. Edit verifications are performed according to the Data Validation Plan. After database lock, the data will be provided as SAS® files to the statistical team for data analysis under the leadership of the trial’s methodologist.

### Data interpretation issues

A steering committee comprising the two co-principal investigators (Dr. F. Jaisser, Dr. S. Girerd), the methodologist of the trial (Dr. N. Girerd), and Prof. P. Rossignol and Prof. L. Frimat will be in charge of the analysis and interpretation of the data as well as manuscript drafting and editing. The sponsor and funders will not have ultimate authority on the decision to submit the report to publication.

## Discussion

### Context of organ shortage and susceptibility of expanded criteria donor grafts to ischemia/reperfusion lesions

The number of patients waiting for KT is increasing at a dramatic pace [22] due to the higher incidence of ESRD [22] (related to the higher prevalence of diabetes and hypertension worldwide as well as to the longer life expectancy of patients with cardiovascular comorbidities) and better access to the waiting list (even for elderly patients or patients with comorbidities [22]). Despite the development of living donor donation, organ shortage is a critical public health problem. As a result, the use of kidney grafts from ECDs has steadily increased over the last decades (45.8% of all kidney grafts from deceased donors in France in 2016 (https://www.agence-biomedecine.fr/annexes/bilan2016/donnees/organes/06-rein/synthese.htm)). The mean age of deceased donors is also steadily increasing (in France it is 56.6 years in 2016 versus 41.5 years in 2000 (https://www.agence-biomedecitne.fr/annexes/bilan2016/donnees/organes/06-rein/synthese.htm)).

However, the functionality of grafts from ECDs is lower than that from standard criteria donors (SCDs) [23]. The more frequently observed DGF in the case of KT from an ECD is most likely one of the factors explaining the lower survival of these grafts [7] and the lower estimated GFR observed at one year (https://www.agence-biomedecine.fr/annexes/bilan2016/donnees/organes/06-rein/synthese.htm). ECD grafts are probably more susceptible to lesions related to IRI. Indeed, IRI leads to DGF [10], and also likely to long-term impairment, in a manner similar to AKI leading to CKD [11, 12].

### Impact of MR antagonists for the prevention of IRI and underlying suspected mechanisms

In addition to its classical localization in the distal nephron (distal tubule and collecting duct), leading to body sodium homeostasis and potassium regulation, MR is expressed in vascular endothelial and smooth muscle cells of interlobar arteries of the kidney as well as in the general vascular bed and the heart [24]. Novel non-classical pathophysiological consequences of MR activation leading to cardiac fibrosis, arterial stiffness, and atherosclerosis have also been described [24]. In the setting of heart failure, MRAs have been proven to dramatically improve patient survival in instances of low ejection fraction and post-myocardial infarction [25].

In preclinical models, MR blockade prevents IRI by improving renal blood flow in instances of ischemia [13–16]. MR activation leads to inflammation and oxidative stress, which in turn lead to vasoconstriction in the acute phase [24] and to tissue fibrosis in the case of sustained chronic activation [24] (i.e., tubulo-interstitial fibrosis in the kidney, cardiac fibrosis in chronic heart failure, arteriosclerosis in the vascular bed). The mechanisms underlying the deleterious effects of MR activation during IRI highlight the critical role of MR-mediated oxidative stress, which results in a specific imbalance of vascular endothelin signaling through a post-translational modification of the vasodilatory endothelin B receptor, leading to its functional inactivation and a sustained decrease in renal blood flow [14, 15]. Moreover, the beneficial effect of MRAs lies in the prevention of low-grade inflammation and on the polarization of macrophages toward an anti-inflammatory M2 repair phenotype [17]. Considering that the infiltration of inflammatory cells early after an AKI episode plays an important role in defining effective versus maladaptive repair [12], this likely explains why short-term MRA administration during the I/R period only is able to prevent the long-term consequences of IRI.

### Justification of the choice of MRA, dosing, and timing of drug administration

Eplerenone was chosen for this study given its much greater selectivity for MR than spironolactone [24]. Moreover, eplerenone is not a prodrug (in contrast with spironolactone); thus, it is anticipated that eplerenone will provide rapid efficacy after administration, which is of crucial importance in this context. Indeed, in the case of KT from a deceased donor, the surgical procedure is usually performed as early as possible upon confirmation of the absence of contra-indications, in order to limit cold ischemia time (CIT). Consequently, the time delay between KT confirmation and the transfer of the patient to the operating room is often very short, which does not allow administering the MRA for a long period prior to the KT. For ethical reasons, it is not conceivable to delay the KT in order to administer the MRA, considering that CIT is one of the main factors leading to DGF [26].

With regard to safety, eplerenone has a shorter half-life (4 h) than spironolactone (> 12 h for active metabolites) and has no active metabolite; thus, it is possible to terminate the treatment with rapid improvement in case of adverse event.

With regard to the timing of the first MRA administration, it was not possible for ethical reasons to initiate the study drug before confirmation of the KT. Nevertheless, in preclinical studies in rodents demonstrating a beneficial effect of MRAs in preventing acute IRI, MRAs were also effective when administered up to 3 h after IRI [13, 14].

With regard to eplerenone posology, a dose of 50 mg/day was selected, since most studies showing a beneficial impact of MRA in CKD for reducing proteinuria utilized a dose of 25 mg/day of spironolactone [27]. Moreover, randomized controlled trials (RCTs) in heart failure after myocardial infarction employed a dose of 25 mg/day of spironolactone or 25–50 mg/day of eplerenone (25 mg/day spironolactone in the Randomized Aldactone Evaluation Study (RALES) [28], 25–50 mg/day eplerenone in the Eplerenone Post-Acute Myocardial Infarction Heart Failure Efficacy and Survival Study (EPHESUS) [29], 25–50 mg/day eplerenone in the Eplerenone in Mild Patients Hospitalization and Survival Study in Heart Failure (EMPHASIS-HF) [30]). Consequently, the administration of 25 mg eplerenone twice a day should be sufficiently effective along with good safety. Indeed, this dose used in EPURE is two to four times lower than the dose (or the equivalent dose of spironolactone) utilized in other studies in patients with kidney disease in which no major/threatening hypotensive or hyperkalemic effect was observed [31, 32].

### Justification of the primary outcome

In this study, we chose to assess graft function at 3 months as opposed to the incidence of DGF. It is indeed very difficult to determine a consensual definition of DGF, considering that 18 definitions have been proposed to date in the literature [9], hence reflecting the complexity of the mechanisms involved in this clinical feature. The most frequent definition is the necessity of at least one dialysis session during the first week following KT. Despite its practical value for data collection (easy to collect), this definition has certain pitfalls.The decision to perform or not perform a dialysis session is the result of the clinician’s assessment and may vary according to the physician. Moreover, a dialysis session may be performed due to hyperkalemia or overhydration, which can be independent or partially dependent of DGF. Furthermore, despite its statistical association with inferior graft survival, it is not well established that DGF per se has direct deleterious consequences on long-term graft survival [9].

Many studies have conversely demonstrated that estimation of GFR at 6 or 12 months post-KT by creatinine measurement was a good predictor of long-term graft survival [33]. Several studies have also demonstrated an association between graft function at 3 months and long-term graft survival [34–37]. For example, a multicenter Spanish study comprising nearly 4500 kidney graft recipients demonstrated that graft function at 3 months was a major determinant of the slope of GFR decline during long-term follow-up [36].

In the present study, graft function at 3 months post-KT measured by iohexol clearance was chosen as primary outcome. In our view, this 3 months timepoint is (1) sufficiently long to represent a valid predictor of long-term graft survival and (2) sufficiently close to the time of KT to accurately assess the impact of the intervention in preventing IRI lesions without being modified by other events (graft rejection, toxicity of calcineurin inhibitors).

Moreover, in kidney graft recipients, the estimation of GFR by formulas validated in CKD (for example, the Modification of Diet in Renal Disease (MDRD) formula [38]) either underestimate or overestimate the true GFR, particularly in the case of low graft function [39]. Therefore, it is recommended to preferentially use a reference method of GFR measurement [39, 40]. Iohexol clearance was thus chosen herein, since it is easy to perform, and has a shorter measurement time and is less costly but with similar accuracy in comparison to other reference techniques such as inulin clearance or (51)Cr-EDTA clearance, even in the case of advanced CKD [41, 42].

### Safety concerns

The administration of an MRA in the setting of ESRD among patients receiving a kidney graft with high risk of DGF raises the question of the risk of hyperkalemia. Close monitoring of hyperkalemia is necessarily required in this setting. Medical interventions including the use of potassium-binding resins, correction of metabolic acidosis with bicarbonate therapy, insulin therapy, or beta-2 mimetic is allowed in the study protocol. Supplementary dialysis for hyperkalemia may also be advisable in some patients. Nevertheless, dialysis requirement is very frequent in the case of KT with ECD, since DGF (defined by the necessity of at least one dialysis session in the fourth week post-KT) is observed in 25% of KT with ECD according to French data (https://www.agence-biomedecine.fr/annexes/bilan2016/donnees/organes/06-rein/synthese.htm). Of note, only those patients on chronic hemodialysis before KT are included in this trial, in order to be able to easily schedule a dialysis session in the event of hyperkalemia without the necessity to place a central dialysis catheter specifically for this purpose, which could raise certain ethical issues.

Moreover, following the rationale of the study, one can anticipate that the prevention of IRI by MRA administration will lead to a reduction in DGF and consequently a lower risk of hyperkalemia in the early post-operative period. Finally, given the short half-life of eplerenone (4 h) and the short administration period (4 days), the risk of hyperkalemia will be very transitory and should be easily manageable in the setting of usual acute transplant unit care.

Finally, note that studies in CKD with MRA (whether in dialysis [43] or not [44]) reported a very limited effect of MRA on blood pressure. Therefore, we do not anticipate a major risk of hypotension that could slow or impede graft recovery.

### Clinical implications

Our hypothesis is that MR blockade in the perioperative period of KT will improve the short-term graft function of ECD graft recipients. Increasing the pool of deceased donor organs is a public health necessity considering the current worldwide organ shortage. ECDs are an important source of grafts (https://www.agence-biomedecine.fr/annexes/bilan2016/donnees/organes/06-rein/synthese.htm) [22]; however, optimizing graft function is crucial in order to further optimize the clinical results obtained with this type of transplant. Optimizing graft function is also a priority in instances of KT with graft from non-heart beating donors [45], an indication in which MRA could also be tested in the future if the current results of EPURE are positive.

## Trial status

At the time of submission, participating hospitals have been enrolled (seven transplant centers) and randomized, and patient recruitment has begun in six centers.

## Additional files

Additional file 1: SPIRIT 2013 checklist: recommended items to address in a clinical trial protocol and related documents. (DOC 58 kb)

Additional file 2: Informed consent materials 1. (PDF 189 kb)

Additional file 3: Informed consent materials 2. (PDF 142 kb)

## Abbreviations

ACEi: Angiotensin-converting enzyme inhibitor
AKI: Acute kidney injury
ARB: Angiotensin receptor blocker
BP: Blood pressure
CKD: Chronic kidney disease
DGF: Delayed graft function
ECD: Expanded criteria donor
eGFR: Estimated glomerular filtration rate
ESRD: End-stage renal disease
GFR: Glomerular filtration rate
I/R: Ischemia/reperfusion
IRI: Ischemia/reperfusion injury
KT: Kidney transplantation
MR: Mineralocorticoid receptor
MRA: Mineralocorticoid receptor antagonist
RCT: Randomized controlled trial
