# Supplementary material for: EPURE Transplant (Eplerenone in Patients Undergoing Renal Transplant) study: study protocol for a randomized controlled trial
Source: Trials. 2018 Oct 30;19:595. doi: 10.1186/s13063-018-2956-1 (PMC6208100; doi:10.1186/s13063-018-2956-1)
Supplement: Supplementary file 2 — Informed consent materials 1. (PDF 189 kb) [file 13063_2018_2956_MOESM2_ESM.pdf]

DOCUMENT D'INFORMATION
Etude EPURE TRANSPLANT (étude principale)

Lisez attentivement ce document et posez toutes les questions qui vous sembleront utiles. Vous pourrez alors décider si vous voulez participer à cette recherche ou non.

Titre de l'étude: Essai randomisé contrôlé en double aveugle contre placebo du blocage précoce du récepteur minéralocorticoïde par l'éplérénone sur la fonction du greffon à 3 mois en transplantation rénale EPURE Transplant (Eplerenone in Patients Undergoing REnal Transplant)

Nom du promoteur : CHRU de Nancy
Adresse du promoteur : 29, avenue du Maréchal de Lattre de Tassigny
54035 Nancy

Madame, Monsieur,

Le docteur (Nom et prénom)…………………………………………………………………
Adresse ………………………………………………………………………………………
Téléphone …………………………
médecin investigateur, vous propose de participer à une étude de recherche clinique.

Avant de décider de participer à cette étude, il est important pour vous d'en comprendre l'objectif ainsi que ses implications. Prenez le temps de lire consciencieusement les informations suivantes. Si toutefois certains points manquent de clarté ou si vous avez besoin d'informations complémentaires, n'hésitez pas à poser toutes les questions nécessaires au médecin de l'étude.

Si vous acceptez de participer à cette étude thérapeutique, vous ne pourrez participer à aucune autre étude thérapeutique durant votre implication dans cette étude (jusqu'à 3 mois post greffe).

Nous vous demanderons de signer un formulaire de consentement. Cette signature confirmera que vous êtes d'accord pour y participer.

1. OBJECTIF DE L'ETUDE

Vous êtes atteint d'une insuffisance rénale terminale ayant nécessité votre mise en hémodialyse chronique et vous allez bénéficier d'une transplantation rénale à partir d'un donneur décédé.
En cas de transplantation d'organe, on sait que la période entre le moment du prélèvement chez le donneur et les quelques jours suivant la transplantation du greffon chez le receveur, est à risque d'entraîner des lésions du greffon. La vitesse de récupération (remise en route) du greffon peut ainsi être plus ou moins rapide. Certains greffons peuvent présenter un retard de reprise de fonction,

nécessitant aussi longtemps que nécessaire (généralement quelques jours) la poursuite de l’hémodialyse, jusqu’à ce que le greffon soit suffisamment efficace pour corriger les anomalies de votre prise de sang et réguler votre état d’hydratation.

Le bon fonctionnement du greffon à long terme est en partie dépendant de la façon dont on parvient à limiter les lésions initiales et à éviter le retard de reprise de fonction. Or, à l’heure actuelle, ces moyens sont limités. Mis à part le fait de conserver de manière optimale le greffon après le prélèvement (au froid ou sur une machine) et le fait de pratiquer le plus rapidement possible la transplantation après le prélèvement du greffon, il n’y a pas de traitement validé permettant de limiter les conséquences délétères liées au prélèvement et à la transplantation.

L’objectif principal de cette étude est de déterminer si la prise d’un médicament, l’éplérénone, pendant 4 jours à partir de la greffe, permet d’améliorer la fonction de votre greffon. En effet, des travaux expérimentaux ont montré que l’administration de médicaments de la même famille que l’éplérénone permettait de considérablement réduire les lésions des reins après diminution de la vascularisation rénale. L’éplérénone appartient à la famille des médicaments bloqueurs des récepteurs de l’aldostérone.

Si vous acceptez de participer à cette ~~l’~~étude, vous serez donc susceptible d’avoir, en plus de vos traitements anti-rejets, un médicament, l’éplérénone, sous forme de gélules. Ce médicament est commercialisé depuis plusieurs années en France et déjà largement utilisé dans une autre indication que la transplantation, chez les patients atteints d’insuffisance cardiaque, après un infarctus, afin de prévenir l’aggravation de leur état cardiaque.

Il est important de savoir que si vous acceptez de participer à cette étude, cela n’influencera en rien le reste des médicaments que vous allez recevoir pour la transplantation, et en particulier les médicaments anti-rejets. Afin de juger de manière objective de l’effet de l’éplérénone, cette étude est réalisée chez deux groupes de patients : un groupe qui recevra l’éplérénone (**groupe « éplérénone »)**, et un groupe qui recevra un placebo (**groupe « placebo »**). Le placebo est une gélule ne contenant pas de médicament.

Cette étude comportant 2 groupes de patients est dite « randomisée », c’est-à-dire que le groupe de l’étude auquel vous appartiendrez ne dépendra ni de vous, ni du médecin de l’étude, mais sera préétablie par tirage au sort de manière informatique. Vous aurez la même probabilité d’appartenir à l’un ou l’autre groupe. Quel que soit le groupe auquel vous appartenez, vous aurez le même suivi. Ni vous, ni votre médecin, ni l’équipe soignante ne sauront quel médicament (éplérénone ou placebo) vous aurez reçu. Ceci permet de rendre l’analyse des résultats la plus objective possible et de déterminer si l’éplérénone est efficace pour améliorer la reprise de fonction du greffon. Cependant, si votre état de santé le nécessite, votre médecin pourra obtenir rapidement votre groupe de traitement (placebo ou éplérénone) auprès du Centre Antipoison de Lyon.

Cette étude concerne 132 patients en France, traités pendant 4 jours et suivi pendant 3 mois.
Cette étude sera réalisée dans des établissements de soins en France métropolitaine.

1. DEROULEMENT DE L'ETUDE

Après une lecture attentive de cette note d'information, si vous acceptez de participer à cette étude, vous signerez le formulaire de consentement éclairé en deux exemplaires dont un vous sera remis.
Si vous répondez aux critères d'inclusion du protocole et une fois que votre médecin sera certain que vous allez bien pouvoir bénéficier de la transplantation rénale, vous prendrez pendant 4 jours consécutifs de l'éplérénone ou du placebo à une dose de 50 mg par jour (soit deux gélules de 25 mg/jour).

Calendrier de l'étude :

Visite d'inclusion :

Après avoir répondu à vos questions et obtenu votre accord écrit, et après avoir vérifié que vous répondez aux critères d'inclusion/non inclusion, nous réaliserons un examen clinique et un interrogatoire médical. Si votre taux de potassium n'a pas été mesuré dans les 12 heures précédentes nous le mesurerons avant la prise du traitement. En fonction du résultat, votre néphrologue pourra décider d'organiser une séance de dialyse avant la greffe.

Prises du médicament :

Vous aurez votre première prise d'éplérénone ou de placebo (25 mg), en même temps que la première prise des traitements anti-rejets, avant le départ pour le bloc opératoire. La seconde prise de médicament (éplérénone ou placebo) aura lieu à environ 12h de la première prise. Les administrations suivantes de l'éplérénone ou du placébo auront lieu toutes les 12 heures ± 2h, à la dose de 25mg, avec 8 prises au total.

Suivi durant 7 jours post transplantation

Une fois transplanté, vous serez suivi pour les besoins de l'étude durant 7 jours consécutifs. Vous serez alors encore hospitalisé, dans le cadre de votre transplantation. Nous réaliserons un examen clinique et un interrogatoire médical chaque jour. Nous vérifierons et recueillerons vos données cliniques (poids, tension artérielle, urines de 24 heures) et biologiques (prise de sang et analyse des urines de 24 heures) issus des examens réalisés dans le cadre du suivi habituel de la transplantation.

Pendant toute la durée de traitement et 48h après la dernière prise, nous mesurerons votre taux de potassium 1 fois par jour en plus des examens habituels dans le cadre de la greffe, afin de nous assurer que vous tolérez bien le traitement de l'étude.

<u>Suivi à 28 jours post transplantation :</u>

Lors de votre visite de suivi habituel à 28 jours post transplantation, nous réaliserons un examen clinique (poids, tension artérielle, urine de 24 heures) et un interrogatoire médical. Nous recueillerons vos données biologiques issues des examens réalisés dans le cadre du suivi habituel de la transplantation (prise de sang et analyse des urines de 24 heures).

<u>Suivi à 3 mois post transplantation :</u>

Lors de votre visite réalisée dans le cadre du suivi habituel à 3 mois post transplantation, nous vérifierons et recueillerons vos données cliniques (poids, tension artérielle, urine de 24 heures) et biologiques issues des examens réalisés dans le cadre du suivi habituel de la transplantation (prise de sang et analyse des urines de 24 heures).

Nous mesurerons également de manière très précise la fonction de votre greffon par une méthode appelée : « clairance à l'iohexol ». Cette méthode est utilisée couramment dans certains centres de transplantation car elle est simple, fiable et non dangereuse pour le greffon. Elle est plus précise que la simple mesure de la créatininémie.

- Elle implique l'injection par voie intra veineuse d'un produit (dit de contraste qui peut rarement susciter des évènements indésirables à type d'allergies) suivie du recueil de 6 échantillons sanguins de 5 ml sur une période de 2h30. Les échantillons sanguins seront rapatriés, des centres vers le Centre de Ressources Biologiques du CHRU de NANCY et seront ensuite expédiés et analysés au laboratoire de Biochimie du Nouvel Hôpital Civil de Strasbourg Une fois les dosages réalisés, le surplus de vos échantillons sanguins sera détruit.

En fonction de l'organisation de votre service, il est possible que vous vous déplaciez dans un service spécialisé (ex : service d'explorations fonctionnelles) ou un centre d'investigation clinique afin de réaliser cet examen.

La durée totale de votre participation sera d'environ 3 mois.

Modalités de prise en charge à la de fin de votre participation

Dans les deux groupes, à l'issue de la fin théorique de participation (suivi à 3 mois post transplantation), ou en cas d'arrêt prématuré de la recherche, vous serez suivi(e) selon les modalités habituelles par votre médecin dans votre centre, cela conformément aux recommandations usuelles.

Suivi à long terme

Afin de connaître les potentiels effets à long terme du médicament étudié, nous interrogerons la base de données CRISTAL de l'Agence de Biomédecine. La consultation de cette base de données permettra de connaître l'évolution de votre état de santé et l'évolution de votre greffon à 1 an, 3 ans et 10 ans de la transplantation. Ceci ne nécessitera aucune visite médicale supplémentaire, puisque ces données médicales sont collectées de manière habituelle par l'équipe médicale de transplantation qui va vous suivre.

Collections biologiques :

Nous vous informons que les échantillons sanguins prélevés durant la visite de suivi à 3 mois post transplantation pour la mesure de la fonction de votre greffon (« clairance à l'iohexol ») feront l'objet d'une collection biologique. Vos échantillons sanguins seront ainsi rapatriés vers le CHRU de Nancy et seront conservés jusqu'à la fin de l'étude au sein du Centre de Ressources Biologiques Lorrain (CRB Lorrain, CHRU Nancy, 4 rue du Morvan, 54500 Vandoeuvre les Nancy), sous la responsabilité du Prof Patrick ROSSIGNOL.

A la fin de l'étude, vos échantillons sanguins seront ensuite expédiés au laboratoire de Biochimie du Nouvel Hôpital Civil de Strasbourg qui réalisera les mesures de la fonction de votre greffon (« clairance à l'iohexol »).

Une fois les dosages réalisés, le surplus de vos échantillons sanguins sera détruit.

Par ailleurs, lors des visites de suivi dans le cadre de l'étude et en fonction des possibilités techniques de votre centre, nous vous proposons de participer à l'élaboration d'une seconde collection biologique. Cette collection sera constituée à l''inclusion (juste avant la transplantation) puis à 1 jour, 7 jours, 28 jours et 3 mois de la transplantation afin de mesurer ultérieurement des constituants du sang (plasma, sérum). Un volume de 24, 5 mL de sang supplémentaire répartis en tubes sera prélevé et environ 15 mL d'urines seront recueillies lors de ces 5 journées.

Cette collection sera stockée sous la responsabilité du Professeur Patrick ROSSIGNOL, au sein du CRB Lorrain, 4 rue du Morvan, 54500 Vandoeuvre les Nancy. Elle sera conservée jusqu'à épuisement et pourra faire l'objet d'études ultérieures sur les pathologies et les effets du traitement étudiés dans le protocole EPURE Transplant.

Au cours ou à l'issue de la recherche, et sauf opposition de votre part, vos échantillons biologiques et vos données médicales associées dé-identifiées (c'est-à-dire codées, sans mention de votre nom et prénom) pourront être transmis et utilisés par des équipes de recherche nationales et internationales conformément aux réglementations en vigueur pour des analyses complémentaires dans le même domaine de recherche c'est-à-dire les maladies cardio-rénales et la transplantation.

2. BENEFICES EVENTUELS DE L'ETUDE

Vous pourrez ou non retirer des bénéfices de votre participation à cette étude.

Le bénéfice attendu lié au traitement par éplérénone est l'amélioration de la reprise de la fonction du greffon avec une vitesse de récupération plus rapide, et éventuellement de réduire la durée d'hospitalisation.

A moyen terme, on attend une meilleure fonction du greffon à 3 mois, qui elle-même est associée à une meilleure qualité de vie. Dans la mesure où la fonction du greffon à 3 mois prédit également la fonction du greffon à long terme, et sa durée de fonctionnement, on anticipe un allongement de la durée de fonctionnement du greffon.

En cas de résultats positifs de l'étude, c'est-à-dire si il est démontré que l'éplérénone est efficace pour améliorer la fonction des greffons à 3 mois, cela pourrait modifier les recommandations concernant la prise en charge des patients transplantés.
Par ailleurs, votre participation à cette étude permettra d'améliorer les connaissances sur les mécanismes impliqués dans la reprise de fonction des greffons et pourra améliorer la prise en charge des transplantés.

3. RISQUES EVENTUELS DE L'EPLERENONE

L'administration de l'éplérénone peut entrainer la survenue d'une augmentation du taux de potassium dans votre sang durant la durée de traitement, nécessitant soit des traitements médicamenteux, soit si besoin le recours à l'hémodialyse. Il est important de savoir que la nécessité éventuelle de recourir à la dialyse même après la greffe ne modifie pas la reprise de fonction du greffon.

Ce risque cessera à la fin du traitement (dont la durée totale est de 4 jours seulement). Une surveillance accrue du taux de potassium sera nécessaire jusque 48h après la dernière prise, et entraînera au moins six prélèvements sanguins supplémentaires de 5 ml par rapport aux soins habituels liés à la transplantation.

Par ailleurs, l'administration d'éplérénone est à risque d'entraîner une diminution modérée de votre pression artérielle, qui sera donc surveillée.

Les évènements indésirables suivants sont possibles en cas de traitement par Eplérénone et sont réversibles à l'arrêt du traitement. La durée du traitement dans cette étude étant courte par rapport à la durée pendant laquelle est habituellement administrée l'éplérénone, il est attendu que ces effets soient beaucoup plus rares que ceux décrits ci-dessous (tableau des effets secondaires rapportés dans le cas de l'utilisation habituelle de l'éplérénone).

**Evénements indésirables fréquents > 1/100, < 1/10**	**Evènements indésirables peu fréquents > 1/1000, < 1/100**	**Evènements indésirables Fréquence indéterminée**
Etourdissements, syncope.<br>Infarctus du myocarde.<br>Hypotension.<br>Toux.<br>Diarrhées, nausées, constipation.<br>Éruption cutanée, prurit.<br>Spasme musculaire, douleurs musculo-squelettiques.<br>Élévation de l'urée dans le sang.<br>Infection<br>Hyperkaliémie<br>Insuffisance rénale	Pyélonéphrite, pharyngite Éosinophilie<br>Hypothyroïdie.<br>Hyponatrémie, déshydratation, hypercholestérolémie, hypertriglycéridémie.<br>Insomnie.<br>Céphalées, hypoesthésie.<br>Insuffisance cardiaque gauche, fibrillation auriculaire, tachycardie<br>Thrombose artérielle des membres, hypotension orthostatique.<br>Vomissements, flatulences.<br>Hyperhidrose.<br>Douleurs dorsales.<br>Cholécystite.<br>Gynécomastie.<br>Asthénie, malaise.<br>Élévation de la créatininémie, Diminution des récepteurs du facteur de croissance épidermique,<br>Augmentation de la glycémie	Œdème de Quincke.

Cette étude peut comporter des risques inconnus ou imprévisibles ou encore des complications différentes de celles mentionnées ci-dessus. Pour cette raison, vous serez surveillé(e) très régulièrement par votre médecin investigateur.

Si vous remarquez la survenue d'un effet indésirable durant votre participation, n'oubliez pas d'en informer un des médecins investigateurs.

4. PARTICIPATION VOLONTAIRE

Votre participation à cette étude est entièrement volontaire et bénévole.

Vous êtes libre de refuser d'y participer ainsi que de mettre un terme à votre participation à l'étude à n'importe quel moment, sans encourir aucun préjudice et sans que cela n'entraîne de conséquences sur la qualité des soins qui vous seront prodigués.
Dans ce cas, vous devez informer le médecin investigateur de votre décision.
Dans le cas où vous retirez votre consentement, c'est-à-dire où vous retirez votre accord écrit de participation à cette étude, vous aurez la possibilité de vous opposer à l'exploitation des données recueillies.
Les Autorités de Santé, votre médecin investigateur ou le promoteur peuvent décider de mettre un terme à votre participation à l'étude à n'importe quel moment sans votre consentement préalable. Si cela devait se produire, vous en serez averti et les raisons vous seraient expliquées.
Le Promoteur prend en charge les frais supplémentaires liés à d'éventuels fournitures ou examens spécifiquement requis pour cette recherche.

5. OBTENTION D'INFORMATIONS COMPLEMENTAIRES

Si vous le souhaitez, le Docteur Sophie GIRERD, que vous pourrez joindre au numéro de téléphone suivant : 03.83.15.36.69, pourra répondre à tout moment à toutes vos questions concernant l'étude EPURE Transplant.
A l'issue de l'étude, et à votre demande, vous pourrez être informé(e) des résultats globaux de la recherche par votre médecin investigateur.

6. CONFIDENTIALITE ET UTILISATION DES DONNEES MEDICALES

Dans le cadre de la recherche biomédicale à laquelle le CHRU de Nancy et votre médecin investigateur vous propose de participer, un traitement de vos données personnelles va être mis en œuvre pour permettre d'analyser les résultats de la recherche au regard de l'objectif de cette dernière, qui vous a été présentée.
A cette fin, les données médicales recueillies, y compris les prélèvements sanguins, tout questionnaire et les données relatives à vos habitudes de vie vous concernant, seront transmises au Promoteur de la recherche.
Ces données seront identifiées par un numéro de code et vos initiales.
Le personnel impliqué dans l'étude est soumis au secret professionnel, tout comme votre médecin traitant.
Ces données pourront également, dans des conditions assurant leur confidentialité, être transmises aux autorités de santé.
Conformément aux dispositions de loi relative à l'informatique aux fichiers et aux libertés (loi du 6 janvier 1978), vous disposez d'un droit d'accès et de rectification. Vous disposez également d'un droit d'opposition à la transmission des données couvertes par le secret professionnel susceptibles d'être utilisées dans le cadre de cette recherche et d'être traitées.
Vous pouvez également accéder directement ou par l'intermédiaire d'un médecin de votre choix à l'ensemble de vos données médicales en application des dispositions de l'article L 1111-7 du Code de la Santé Publique.

Ces droits s'exercent auprès du médecin qui vous suit dans le cadre de la recherche et qui connaît votre identité.
Conformément à la réglementation, vos données anonymes recueillies à l'occasion de la présente recherche pourront être transférées et utilisées par des équipes scientifiques nationales ou internationales pour étudier de nouveaux paramètres biologiques pouvant intervenir dans les maladies cardio-rénales et la transplantation.

7. ASSURANCE

Un contrat d'assurance a été souscrit par le promoteur de l'essai, le CHRU de Nancy, auprès de la compagnie d'assurance SHAM, pour couvrir les risques liés à cette étude clinique. Cette assurance couvre la responsabilité du promoteur en tant que promoteur d'une recherche biomédicale et celle de tout autre intervenant, en accord avec l'article L 1121-7 du Code de la Santé Publique.

8. AVIS FAVORABLE DU CPP

Conformément à la loi n°2004-806 du 9 août 2004 relative à la politique de santé publique, le Comité de Protection des Personnes Est III a étudié ce projet de recherche et a émis un avis favorable à sa réalisation le 09/07/2015 .

9. AUTORISATION DE L'ANSM

Conformément à la loi n°2004-806 du 9 août 2004 relative à la politique de santé publique, l'ANSM (Agence Nationale de sécurité du médicament et des produits de santé) et a étudié ce projet de recherche et a émis une autorisation à sa réalisation le 29/06/2015.

FORMULAIRE DE CONSENTEMENT - ETUDE PRINCIPALE

Essai randomisé contrôlé en double aveugle contre placebo du blocage précoce du récepteur minéralocorticoïde par l'éplérénone sur la fonction du greffon à 3 mois en transplantation rénale
EPURE Transplant (Eplerenone in Patients Undergoing REnal Transplant)

Les détails concernant cette étude sont fournis dans la lettre d'information spécifique qui vous a été remise.
Lisez attentivement cette notice et posez toutes les questions qui vous sembleront utiles.
Si vous acceptez de participer à cette étude, veuillez compléter le formulaire ci-dessous.

Nom du Promoteur : CHRU de Nancy
Adresse du promoteur : 29, avenue du Maréchal de Lattre de Tassigny – 54035 NANCY cedex
Je soussigné (e), Mme, M. …………………………………………………… (*nom complet en lettres capitales)* déclare avoir compris le but et les modalités de cette étude, qui m'ont été pleinement expliqués par le Docteur …………………………………………………(*nom complet en lettres capitales)*, médecin investigateur.

J'ai reçu le formulaire d'information spécifique que j'ai eu la possibilité d'étudier avec attention.

Des réponses ont été apportées à toutes mes questions.

J'ai disposé d'un délai de réflexion avant de prendre ma décision.

Je m'engage à ne pas participer à une autre étude thérapeutique pendant toute la durée de l'étude (3 mois post transplantation).

J'accepte de participer à cette recherche dans les conditions précisées dans le formulaire d'information ci-joint. Je demeure libre de quitter l'étude à tout moment sans que cela n'affecte ma prise en charge médicale ultérieure. J'en informerai alors le médecin investigateur.

Le fait de ne plus participer à cette recherche ne portera pas atteinte à mes relations avec mes médecins et ne remettra pas en cause la qualité des soins ultérieurs.

J'ai été informé(e) que conformément à la réglementation sur les études cliniques, le Comité de Protection des Personnes Est III de Nancy a rendu un avis favorable pour la réalisation de cette étude en date du 09/07/2015 et l'ANSM a donné son autorisation pour la réalisation de cette étude en date du 29/06/2015.

J'ai également été informé(e) que conformément à la loi en vigueur, un contrat d'assurance a été souscrit par le promoteur de la recherche.

Toutes les données me concernant, y compris mon dossier médical, resteront confidentielles. Je n'autorise leur consultation que par les personnes qui collaborent à la recherche, aux personnes chargées par le promoteur de contrôler la qualité de l'étude ainsi que par un représentant des autorités de santé.

J'accepte que les données enregistrées à l'occasion de cette recherche puissent faire l'objet d'un traitement informatisé par le promoteur ou pour son compte. J'ai bien noté que le droit d'accès prévu par la loi du 6 janvier 1978 relative à l'informatique, aux fichiers et aux libertés (article 39) s'exerce à tout moment auprès du médecin investigateur qui me suit dans le cadre de la recherche et qui connaît mon identité. Je pourrai exercer mon droit de rectification et d'opposition auprès de ce même médecin qui contactera le promoteur de la recherche.

J'accepte que les données enregistrées à l'occasion de cette recherche, les échantillons sanguins et leurs données associées puissent être transférés de manière dé-identifiée (c'est-à-dire de manière codée, sans mention de votre nom et prénom) à d'autres équipes de recherches partenaires

nationales ou internationales. J'ai bien compris qu'aucune recherche ayant une finalité autre que les maladies cardio-rénales et la transplantation ne sera réalisée.

J'atteste être affilié(e) à un régime de sécurité sociale.

Je donne mon consentement pour participer à cette recherche.

Je pourrai à tout moment demander toute information complémentaire au médecin investigateur.

Mon consentement ne décharge en rien l'investigateur et le promoteur de l'ensemble de leurs responsabilités et je conserve tous mes droits garantis par la loi.

Je comprends que j'ai le droit d'être informé(e) des résultats globaux de cette recherche à l'issue de celle-ci. Ils pourront m'être communiqués par courrier en m'adressant au Dr Fréderic JAISSER, Centre d'Investigation Clinique Plurithématique - CHU Nancy - INSERM, Hôpitaux de Brabois, rue du Morvan, 54500 Vandoeuvre les Nancy (tel : 03 83 15 73 22) si je le désire.

**A REMPLIR PAR LE PATIENT**
Nom complet (en lettres capitales) : ……………………………….. <br>Date : ……… <br>Signature du patient :

**A REMPLIR PAR LE MEDECIN INVESTIGATEUR**	
Je soussigné Docteur ……………………… (nom complet en lettres capitales) confirme avoir pleinement expliqué au patient le but et les modalités de cette étude ainsi que ses risques potentiels. Je m'engage à faire respecter les termes de ce formulaire de consentement, conciliant le respect des droits et des libertés individuelles et les exigences d'un travail scientifique.	
Signature du médecin investigateur :	Date : ……………………………..

Fait en deux exemplaires dont l'un sera conservé par l'investigateur et l'autre remis au patient.
