# Supplementary material for: EPURE Transplant (Eplerenone in Patients Undergoing Renal Transplant) study: study protocol for a randomized controlled trial
Source: Trials. 2018 Oct 30;19:595. doi: 10.1186/s13063-018-2956-1 (PMC6208100; doi:10.1186/s13063-018-2956-1)
Supplement: Supplementary file 3 — Informed consent materials 2. (PDF 142 kb) [file 13063_2018_2956_MOESM3_ESM.pdf]

DOCUMENT D'INFORMATION

Etude EPURE TRANSPLANT (étude génétique)

Lisez attentivement ce document et posez toutes les questions qui vous sembleront utiles. Vous pourrez alors décider si vous voulez participer à cette recherche ou non.

Titre de l'étude: Essai randomisé contrôlé en double aveugle contre placebo du blocage précoce du récepteur minéralocorticoïde par l'éplérénone sur la fonction du greffon à 3 mois en transplantation rénale **EPURE Transplant** (Eplerenone in Patients Undergoing REnal Transplant)

Nom du Promoteur : CHRU de Nancy

Adresse du promoteur : 29, avenue du Maréchal de Lattre de Tassigny
54035 NANCY cedex

Madame, Monsieur,

Vous avez accepté de participer à l'étude EPURE Transplant citée ci-dessus,

Le docteur (Nom et prénom)…………….……………………………………………………

Adresse………………………………………………………………………………………..

Téléphone…………………………

médecin investigateur, vous propose de participer à l'étude génétique complémentaire à l'étude EPURE Transplant.

Cette étude va être proposée à tous les patients qui ont accepté de participer à l'étude principale dans les centres participants à la constitution d'une banque d'échantillons de sang et d'urines pour l'étude. Pour réaliser cette étude complémentaire, nous devons recueillir votre consentement. Après une lecture attentive de cette lettre d'information, si vous acceptez de participer, vous signerez le formulaire de consentement éclairé (en 2 exemplaires dont 1 vous sera remis).

Lors de votre inclusion dans l'étude principale EPURE Transplant, puis à 1 jour, 7 jours, 28 jours et 3 mois post transplantation, un échantillon d'urine d'environ 15 ml et un volume de 24,5 ml de sang supplémentaire est recueilli lors de chacune de ces journées. Si vous acceptez de participer à cette étude de génétique, nous pourrons également utiliser une partie de ces tubes de sang et de vos échantillons d'urines pour extraire de l'acide ribonucléique (ARN) et de l'acide désoxyribonucléique (ADN).

Si vous refusez de participer à cette étude de génétique, nous n'utiliserons pas une partie de ces tubes de sang et de vos échantillons d'urines pour cette étude génétique.

Finalité des analyses génétiques prévues par l'étude :

Les analyses génétiques complémentaires ont pour objectif d'étudier dans le sang et les urines, à partir de l'ARN et de l'ADN, les modifications de l'ADN et de nouveaux marqueurs génétiques (en fonction de l'évolution des connaissances dans ce domaine) impliqués dans l'évolution des processus inflammatoires, fibrotiques et lésionnels qui interviennent dans la reprise de fonction des greffons après transplantation rénale.

Vos échantillons dé-identifiés d'ARN et d'ADN (c'est-à-dire codés, sans mention du nom et prénom) seront conservés plusieurs années jusqu'à épuisement sous la responsabilité du Professeur Patrick Rossignol au sein du Centre de Ressources Biologiques Lorrain (CRB Lorrain) CHRU Nancy, 4 rue du Morvan, 54500 Vandoeuvre-les-Nancy.

Au cours et à l'issue de la recherche, et sauf opposition de votre part, vos échantillons d'ARN et d'ADN et vos données médicales associées dé-identifiées (c'est-à-dire codées, sans mention de votre nom et prénom) pourront être transmis et utilisés par des équipes de recherche nationales et internationales conformément aux réglementations en vigueur pour des analyses complémentaires ayant la même finalité.

Aucune autre recherche ou analyse ayant une finalité autre que celle prévue par l'étude ne sera réalisée.

Toute autre recherche ou analyse génétique ayant une finalité autre que celle prévue dans le présent document d'information, mais se rattachant à cette étude et utilisant ses données, sera soumise à l'avis d'un comité de protection des personnes, vous sera expliquée et ne sera réalisée qu'après obtention de votre consentement écrit.

Il n'y a pas de bénéfice direct attendu pour vous mais cette recherche permettra de mieux connaître les marqueurs génétiques impliqués dans l'évolution des processus inflammatoires, fibrotiques et lésionnels qui interviennent dans la reprise de fonction des greffons après transplantation rénale au cours de l'insuffisance rénale.
Il n'y a pas de risques ou de désagréments supplémentaires pour cette étude complémentaire par rapport à l'étude principale.

Votre participation à cette étude complémentaire est entièrement volontaire et un éventuel refus pour cette partie complémentaire ne remet pas en cause votre participation pour le reste de l'étude.
Vous avez le droit de refuser de participer ou si vous acceptez, de retirer votre consentement à tout moment sans encourir aucune responsabilité ni aucun préjudice de ce fait.
Toute nouvelle information devenant disponible au cours de l'étude et pouvant avoir des implications sur votre personne vous sera fournie par le médecin investigateur.

FORMULAIRE DE CONSENTEMENT

EPURE Transplant (étude génétique)

Les détails concernant cette étude sont fournis dans le document d'information spécifique qui vous a été remis.

Lisez attentivement ce document et posez toutes les questions qui vous sembleront utiles.

Si vous acceptez de participer à cette étude, veuillez compléter le formulaire ci-dessous**.**

Titre de l'étude : Essai randomisé contrôlé en double aveugle contre placebo du blocage précoce du récepteur minéralocorticoïde par l'éplérénone sur la fonction du greffon à 3 mois en transplantation rénale. **EPURE Transplant** (Eplerenone in Patients Undergoing REnal Transplant)**.**

Nom du Promoteur : CHRU de Nancy

Adresse du promoteur : 29, avenue du Maréchal de Lattre de Tassigny
54035 NANCY cedex

Je soussigné (e), M…………………………………………………. (*nom complet en lettres capitales)* déclare avoir compris les modalités de l'étude génétique complémentaire qui m'ont été pleinement expliquées par le Docteur ………………………………………………….., médecin investigateur.

Cette étude génétique a pour but d'étudier les modifications de l'ADN et de nouveaux marqueurs génétiques (en fonction de l'évolution des connaissances dans ce domaine) impliqués dans l'évolution des processus inflammatoires, fibrotiques et lésionnels qui interviennent dans la reprise de fonction des greffons après transplantation rénale

J'ai reçu le formulaire d'information spécifique à l'étude génétique que j'ai eu la possibilité d'étudier avec attention. Des réponses ont été apportées à toutes mes questions.

J'ai disposé d'un délai de réflexion avant de prendre ma décision.

J'accepte de participer à cette étude génétique dans les conditions précisées dans le formulaire d'information ci-joint. Je demeure libre de retirer mon consentement à tout moment sans encourir aucune responsabilité ni aucun préjudice de ce fait. J'en informerai alors le médecin investigateur.

Le fait de ne plus participer à cette étude génétique ne portera pas atteinte à mes relations avec mon médecin et ne remettra pas en cause la qualité des soins ultérieurs.

Je pourrai à tout moment demander toute information complémentaire au médecin investigateur.

Mon consentement ne décharge en rien l'investigateur et le promoteur de l'ensemble de leurs responsabilités et je conserve tous mes droits garantis par la loi.

J'accepte que mes échantillons d'ARN, d'ADN et mes données médicales associées dé-identifiées (c'est-à-dire codées, sans mention de mon nom et prénom) puissent être transmises et utilisées par des équipes de recherche nationales et internationales conformément aux réglementations en vigueur et qu'**aucune autre recherche ou analyse ayant une finalité autre que celle prévue par l'étude ne sera réalisée.**

J'accepte que les données enregistrées à l'occasion de cette recherche comportant des données génétiques puissent faire l'objet d'un traitement informatisé par le promoteur ou pour son compte. J'ai bien noté que le droit d'accès prévu par la loi du 6 janvier 1978 relative à l'informatique, aux fichiers et aux libertés (article 39) s'exerce à tout moment auprès du médecin qui me suit dans le cadre de la recherche et qui connaît mon identité. Je pourrai exercer mon droit de rectification et d'opposition auprès de ce même médecin qui contactera le promoteur de la recherche.

Je donne mon consentement pour participer à cette étude génétique complémentaire.

A REMPLIR PAR LE PATIENT	
Nom complet (en lettres capitales) : ……………………………….. Date : ………………………… Signature du patient :	
A REMPLIR PAR L'INVESTIGATEUR	
Je soussigné Docteur ………………………………….……. (*nom complet en lettres capitales*) confirme avoir pleinement expliqué au patient le but et les modalités de cette étude génétique ainsi que ses risques potentiels. Je m'engage à faire respecter les termes de ce formulaire de consentement, conciliant le respect des droits et des libertés individuelles et les exigences d'un travail scientifique.	
Signature de l'investigateur :	Date : ……………………………..
